# Sunken eyes as a peculiar finding in neuronal ceroid lipofuscinoses

**DOI:** 10.1055/s-0046-1815957

**Published:** 2026-02-27

**Authors:** Raphael Pinheiro Camurugy da Hora, Victor Rebelo Procaci, João Victor Cabral Correia Férrer, Thiago Yoshinaga Tonholo Silva, Flávio Moura de Rezende Filho, José Luiz Pedroso, Orlando Graziani Povoas Barsottini

**Affiliations:** 1 Universidade Federal de São Paulo, Escola Paulista de Medicina, Departamento de Neurologia, São Paulo SP, Brazil.

**Figure 1:**
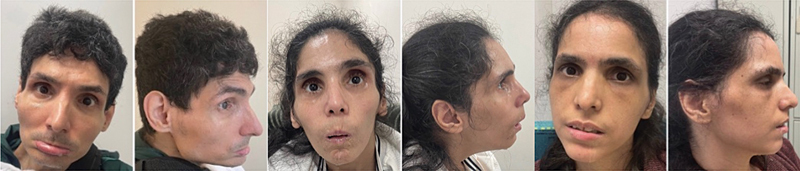
Frontal and lateral photograph of each patient showing the characteristic peculiar feature of sunken eyes. The patient's eyes appear deeply set within the orbital sockets, with noticeable shadowing and loss of soft-tissue volume around the eyes.

We describe three siblings from a Brazilian family with neuronal ceroid lipofuscinoses (NCL) type 2, all presenting with pronounced sunken eyes alongside other typical manifestations: progressive ataxia, seizures, cognitive decline, and motor impairment 1 ( Figure 1 ). Magnetic resonance imaging revealed olivopontocerebellar atrophy in all patients. Genetic testing identified a pathogenic variant in the TPP1 gene in one sibling.

Neuronal ceroid lipofuscinoses are a group of progressive neurodegenerative disorders caused by genetic mutations leading to lysosomal dysfunction. 2 While the core symptoms are well documented, sunken eyes as a clinical feature is rarely reported. We postulate this sign in NCLs may reflect disease severity or complications such as malnutrition, thus warranting further investigation.
